# Nanocrystallized Oleanolic Acid Better Inhibits Proliferation, Migration and Invasion in Intracranial Glioma via Caspase-3 Pathway

**DOI:** 10.7150/jca.38847

**Published:** 2020-01-29

**Authors:** Ruicheng Fan, Heng Wang, Liyuan Zhang, Teng Ma, Yanping Tian, Hongli Li

**Affiliations:** 1 Department of Histology and Embryology, College of Basic Medicine, Army Medical University, Chongqing, China.; 2 Battalion 3, College of Basic Medicine, Army Medical University, Chongqing, China.; 3 Battalion 4, College of Basic Medicine, Army Medical University, Chongqing, China.

**Keywords:** oleanolic acid, nano-particles, glioma cells, migration and invasion abilities, lipophilicity

## Abstract

Glioma associates with high malignancy and poor prognosis for traditional treatment. Oleanolic acid (OA) has been confirmed to have an inhibitory effect on different kinds of tumors, while accompanying with low efficiency because of its large molecular mass and low solubility. Nanoliposome is an appropriate drug delivery system that can compensate for the limitations of traditional insoluble drugs, involving improvement of their solubility, stability and lipophilicity. In the present study, we comprised of OA covered with nanoliposomes, named OA^nano^, to observe antitumor effects on U87 glioma cells. The results showed that OA^nano^ raised the solubility and oil-water partition coefficient. OA^nano^ suppressed proliferation of U87 glioma cells, and also had an anticancer effect on U87 glioma cells, which was found to be higher than that of OA. Moreover, treatment with OA^nano^ induced apoptosis and degraded migration ability by caspase-3 pathway. In conclusion, our results demonstrated that OA covered with nanoliposomes led to enhanced anticancer effects by suppressing proliferation, migration and invasion abilities. The findings may provide a reliable reference for development of new anti-cancer drugs.

## Introduction

Glioma is the most common primary malignant tumor originated in the central nervous system (CNS), accounting for 40% of intracranial tumors 1. It is classified into four grades according to the criteria defined by the World Health Organization (WHO), of which Glioblastoma is highly proliferative and aggressive that often invades the surrounding brain parenchyma, resulting in the dismal prognosis for surgical resections 2-6. Traditional chemotherapy drugs (e.g., temozolomide) mainly causes chemoresistance and remains ineffective with recurrent glioblastoma 5, 7, 8.

Oleanolic acid (OA) is a class of pentacyclic triterpenoids extracted from natural medicines, such as the fruits of *Ligustrum lucidum*, and the whole grass of scutellariae 4. It is a 3β-hydroxyolean-12-en-28-oic acid. The chemical equation of OA is C_30_H_48_O_3_ and its molecular mass is 456.71 9. Previous studies have indicated antitumor activity of OA, for instance, Wang *et al*. verified that OA promotes the apoptosis of HL-60 promyelocytic leukemia cells by up-regulating the expression level of* cav-1* 10; Abdelmageed *et al*. confirmed that the Oleanolic acid methylester (OAME) has a concentration-dependent anti-proliferative effect on PC-3 prostate cancer cells through down-regulating the expression level of Ki-67 9. However, previous studies concerning intracranial tumors have faced with a challenge of OA's poor solubility 11, 12. Hence, whether it has a similar effect on glioma cells has still remained elusive.

It also has been reported that hydrophobicity can enhance circulation time and endocytosis 13, 14. Uncontrolled tumor cell proliferation and incomplete vascularization lead to formation of tumors ranging in size from 100 nm to 1 μm, which can be easily permeated by nanoliposomes. Additionally, poor lymphatic drainage increases the retention and permeation, providing selective retention release by nanoliposomes 14. Thus, nanoliposomes are not only commercially approved as a product for cancer therapy, but also can theoretically assist OA to enhance its solubility and specificity. Hence, OA^nano^ was constructed to observe antitumor effects on U87 glioma cells in the present study. Our results demonstrated that OA^nano^ led to enhanced anticancer effects by suppressing proliferation, migration and invasion abilities. The findings may provide a reliable reference for development of new anti-cancer drugs.

## Materials and Methods

### Materials

The human GBM cell lines U87-MG (male glioblastoma, ATCC, # HTB-14) were purchased from American Type Culture Collection (ATCC). The cell line was validated by short tandem repeat profiling analysis by ATCC. U87 cell line that stably expresses luciferase (U87-Luc) was established through lentiviral transduction. Transduced colonies (bioluminescent U87 cells, or U87-Luc) were selected with 5 μg/mL blasticidin and examined for luciferase expression by detecting its activity with D-Luciferin. U87 and U87-Luc cells were cultured in Dulbecco's modified Eagle's medium (DMEM; Hyclone, Logan, UT, USA) /high glucose supplemented with 10% fetal bovine serum (FBS, Gibco, Carlsbad, CA, USA) in 5% CO_2_ at 37°C.

BALB/c nude mice were purchased from pathogen-free Animal Center of the Army Medical University (Chongqing, China) and were 6-8 weeks with either sex. All animal experiments were conducted according to the guidelines for laboratory animal care and use, and were approved by the IACUC and the Ethics Committee of the Army Medical University.

TRIZOL reagent was purchased from Invitrogen, USA. Easy-Load™ PCR Master Mix (GREEN, 2X) was purchased from Beyotime, Shanghai, China. Antibodies against Vimentin, Casapase-3, PCNA and Ki-67 were purchased from Boster, Wuhan, China.

### Methods

#### Construction and Observation of OA^nano^

60 mg of phosphorylcholine and 6 mg of cholesterol were dissolved in 10mL of diethyl ether, then 15μL of fluorescent dye (DiI, dissolved in dimethyl sulfoxide at a concentration of 5mol/L) and OA powder was added to the mixture for labeling. 50μL of the lipid solution mixture was dropped on an ITO glass slide and then evaporated under vacuum for 2 hours to form a stable lipid film. The morphology was observed under a scanning electron microscope.

#### Diffusion Test

5mL OA or OA^nano^ was added to 0.1μm microporous filter membrane with buffer of the same volume at (37±0.5)℃. The continuous filtrate was diluted to the appropriate concentration with blank medium, the absorbance value was determined at 254 nm according to spectrophotometry, and the corresponding mole concentration and accumulated diffusion rate was calculated by self-control method. Using the same method 3 times took the average value and drew the dissolution curve.

#### Evaluation of Oil-water Partition Coefficients

Place 4mL ethyl acetate and 4mL water in a separatory funnel, vortex for 20 min, centrifuge for 20 min and then take the supernatant after stratification (the supernatant is a water-saturated ethyl acetate solution, and the rest is an ethyl acetate-saturated aqueous solution). UV scanning in the wavelength range of 200-400 nm shows the maximum absorption wavelength is 254 nm. Weigh a certain amount of OA and OA^nano^ again, add water-saturated ethyl acetate till the concentrations reach 20, 30, 40, 50 or 60 μmol/L, respectively. The absorbance of each solution was measured at 236 nm. The regression equation was A=63.125C+0.0064 (r=0.9999, n=5), A means absorbance and C means concentration. Take 3mL of OA and OA^nano^ solution of different concentrations, add 4mL of saturated solution of ethyl acetate, vortex and centrifuge and then take the supernatant after stratification. Taking ethyl acetate as blank control, the absorbance was measured at 236 nm. The concentration of the aqueous phase OA and OA^nano^ was calculated through regression equation. The ethyl acetate-water partition coefficient of OA and OA^nano^ is K_ow_=C_o_/C_w_.

#### MTT Assay

3-(4, 5-dimethylthiazol-2-yl)-2, 5-diphenyltetrazolium bromide (MTT) assay was used for the measurement of Cell proliferation. U87 cells were plated into 96-well plates and added with 20 μL of MTT (5mg/mL) (Sigma, USA), being incubated for 4h in the 37°C, 5%CO_2_ incubator. And then cells were added with 200μL of DMSO to solubilize the crystals for 20min at room temperature. The optical density was examined with a scanning multi-well spectrophotometer at a wavelength of 490nm.

#### Wound-healing Assay

U87 were incubated in a monolayer in 24-well plates and were seeded to nearly complete confluence. Then the cells were serum starved overnight and then were scratched a wound using a plastic pipette tip. Debris was removed by washing the cells with PBS for three times. The monolayer was cultured in 2% fetal bovine serum -Dulbecco's modified eagle medium (FBS-DMEM) in the 37°C, 5% CO_2_ incubator. 35 μmol/L of OA^nano^ was added in treatment groups (n=3) and the same volume of DMSO was added in control groups (n=3). The photos were taken after 24 h by an inverted microscope (Olympus, IX71, Tokyo, Japan). Healing rates was calculated according to the following equation: HR = (d_1_ - d_2_) / d_1_ * 100% (HR means healing rate, d_1_ means the distance before healing and d_2_ means the distance after healing) .

#### Transwell Migration Assay

U87 cells were serum starved overnight and then were trypsinized and resuspended into DMEM medium. Cells were transferred from serum-free medium to the upper layer of 24-well transwell chamber (Corning, 8-μm pore size) at the concentration of 2 ×10^4^ and DMEM containing 10% FBS was added to the bottom layer. 35 μmol/L of OA^nano^ was added in treatment groups (n=3) and the same volume of DMSO was added in control groups (n=3). After incubating for 24 h, cells on the upper layer (non-migrated) were fixed in 4% methanol for 30min and stained with a 0.1% crystal violet solution for 30min, while bottom cells (migrated) were removed with a cotton swab. Five fields were randomly chosen and the number of non-migrating cells in each field was counted.

#### RT-PCR

U87 cells were treated with OA^nano^ (35μmol/L) or DMSO (same volume of OA^nano^ as control groups) for 24 h. Total RNA was extracted from cells by TRIZOL reagent and the concentration and quality of RNA were measured with Nanodrop Spectrophotometer (IMPLEN GmbH, Munich, Germany). Then, RNA was reversely transcribed into cDNA by using Easy-Load™ PCR Master Mix (GREEN, 2X). The volume of each PCR reaction mixture is 25μL, which is consisted of 12.5μL PCR Master Mix, 1μL sense and antisense primers, 0.5μL cDNA and 10μL Nuclease-Free Water. Then the mixture was run for 35 cycles. Each cycle contains three operations: denaturation at 95°C for 30s, annealing at 55°C for 30s, and extension at 72°C for 60s. GAPDH was used as internal control and RT-PCR was carried out using the following primers: Ki-67 (F: 5'-AAGCCGAAACCAGCTAGACTTTC-3', R: 5'- TGGCGGAGTGGCAACAA -3'), Caspase-3 (F: 5'-TGACTGGAAAGCCGAAACTC-3', R: 5'-AGCCTCCACCGGTATCTTCT-3'), MMP-7(F: 5'-TGTCCTGAATGATACCTATGA-3', R: 5'-TACTCAGTGGATAAAGGTGTA-3'), GAPDH(F: 5'-AGCAAGAGCACAAGAGGAAG-3', R: 5'-GGTTGAGCACAGGGTACTTT-3').

#### Immunohistochemistry (IHC) and Immunofluorescence (IFC)

Glioma tissue was isolated from each group of nude mice (treatment groups treated with OA^nano^ and control groups with DMSO) as described above and fixed by 4% PFA. Then the tissue was dehydrated, embedded in paraffin through standard process (70% ethanol 3 h, 85% ethanol 1 h, 95% ethanol 1 h or overnight, 100% ethanol 1 h x 2, 1/2 ethanol + 1/2 xylene 20 min, xylene 30 min x 2, paraffin I/II/III 1 h respectively) and cut into slices. Sections, which were 4μm thick, were stained with HE and IHC using antibodies against Vimentin. HE and IHC staining results were analyzed by Image proPlus6.0. Another part of Glioma tissue was washed with PBS three times and then fixed by 4% PFA for 0.5h, then permeabilized by 0.1% Triton X-100 for 15min at room temperature and blocked in 5% BSA (Sigma, USA) for 1h. The cells were immunostained with antibody to Caspase-3 (rabbit anti-human Caspase-3, 1:200) at 4°C overnight and then incubated with the secondary antibody (goat anti-rabbit, Boster, Wuhan, China) for 0.5h at room temperature, exposing to no light as possible. The nuclei were counterstained with DAPI. The photos were taken by using a fluorescence microscope (Olympus IX53/DP80, Japan).

#### Xenograft Tumor Formation and Bioluminescence Imaging

Experiments with nude mice were conducted strictly according to the guidelines for laboratory animal care and use, and were approved by the IACUC and the Ethics Committee of the Army Medical University. With a density of 5×10^6^/each point, 10 μL U87-Luc cells were injected in lateral ventricle of nude mice to generate xenografts tumor model. Mice were randomized into DMSO control (n=18) and OA^nano^ treatment groups (n=18). OA^nano^ was administered via intravenous injection every day for 5 days at a dose of 17.5mM/kg, and the control groups received an equal volume of DMSO.

The tumor size which was represented by luminescence was monitored on day 7, day 14 and day 21, using bioluminescence imaging: mice were first anesthetized with isoflurane and then injected intraperitoneally with D-luciferin (120 mg/kg). After ten minutes, bioluminescent signals of the tumors were recorded by Xenogen IVIS System. Mice were sacrificed on day 21 and tumor tissue was extracted. The tumors were measured with a vernier caliper and the volume was calculated as length×width^2^/2, where length means the largest tumor diameter and width means the perpendicular tumor diameter.

#### Statistical Analysis

The results were expressed as mean±SD (standard deviation) and each was repeated at least three times. The t-tests and χ^2^ were used to evaluate differences between the dosing groups and the control groups. Values of P<0.05 were considered statistically significant. All statistical analysis was performed by Graphpad Prism 6 and iPP 5.

## Results

### Construction of OA^nano^ and evaluation of its therapeutic effects

**Figure 1:**
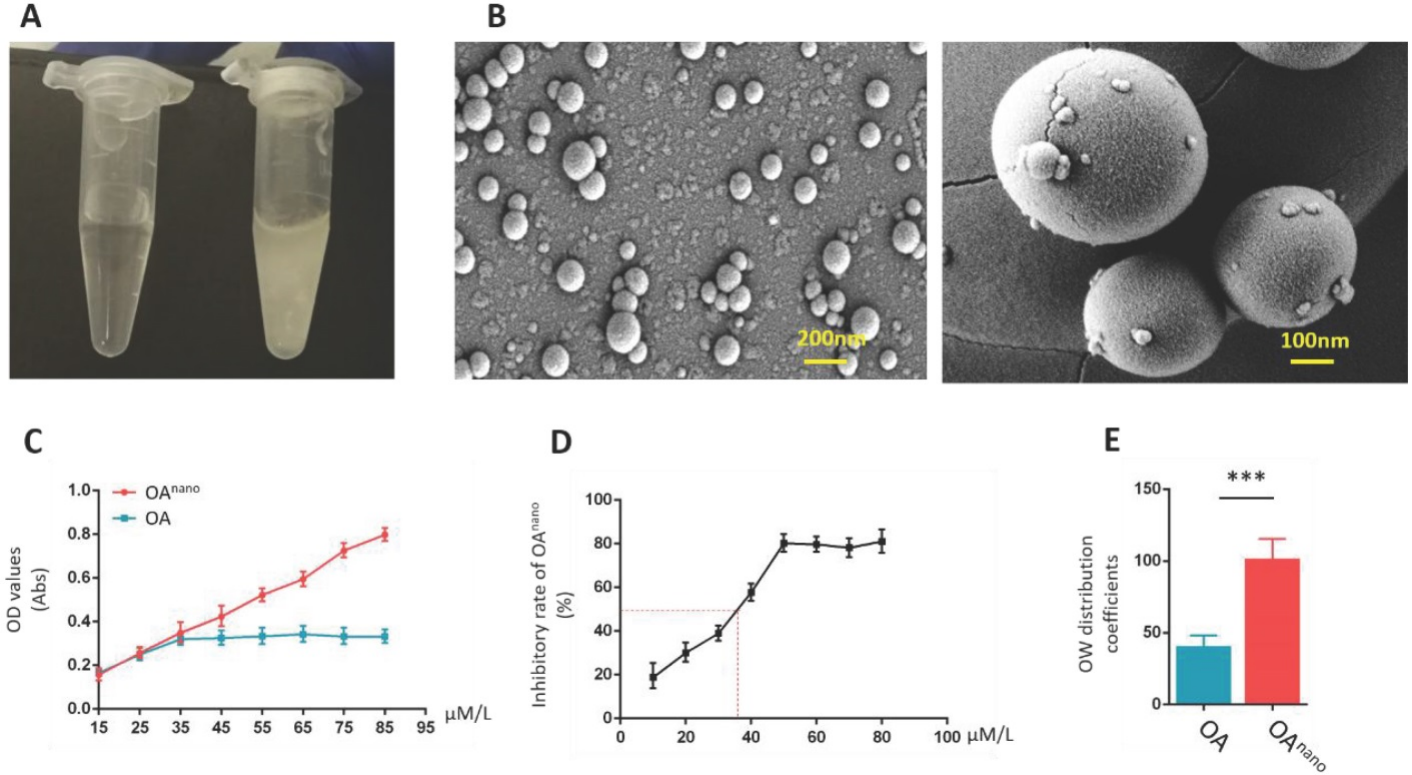
** Construction of OA^nano^ and evaluation of its therapeutic effects. A.** Appearance of DMSO-OA^nano^ (left) and DMSO-OA (right) solution (50 μmol/L). **B.** OA^nano^ was observed under scanning electron microscopy. **C.** Dissolution rates of OA and OA^nano^. **D.** IC_50_ of OA^nano^ (35 μmol/L) indicated by the inhibitory rate. **E.** Oil-water partition coefficients of OA and OA^nano^.

Covered with polymeric nano-particles, OA^nano^-dimethyl sulfoxide (DMSO) solution presented a clear appearance at 50 μmol/l (Fig. 1-A, left), while limited due to its hydrophobic characteristics, and normal OA-DMSO solution was turbid and milky at the same concentration (Fig. 1-A, right). The dissolution rate of OA^nano^ was 2.5-fold higher than normal OA (Fig. 1-C). With the help of scanning electron microscopy (SEM), OA^nano^ could be observed as pure spherical particles, which were evenly distributed (Fig. 1-B) estimated by SEM. The oil-water partition coefficient of OA^nano^ was 101.75, which was about 2-fold higher than that of normal OA (Fig 1-E).

In the current study, U87 glioma cells were treated with OA^nano^ or normal OA at different concentrations (10, 20, 30, 40, 50, 60, 70, 80 μmol/L) during designated time period to verify their therapeutic effects. In OA^nano^ group, 50% of U87 glioma cells lost their activity (IC_50_) at 35 μmol/L (Fig. 1-D), and the following experiments were conducted at this certain concentration. Additionally, when the concentration reached 35 μmol/L, proliferation of U87 glioma cells in OA^nano^ group was lower than that in OA group, which became even more notable when reached 45 μmol/L (Fig. 1-D), which indicated that polymeric nano-particles can improve the effective concentration of OA, so as to emerge a more robust anti-tumor effect.

### OA^nano^ suppresses the growth of U87 glioma cells

**Figure 2:**
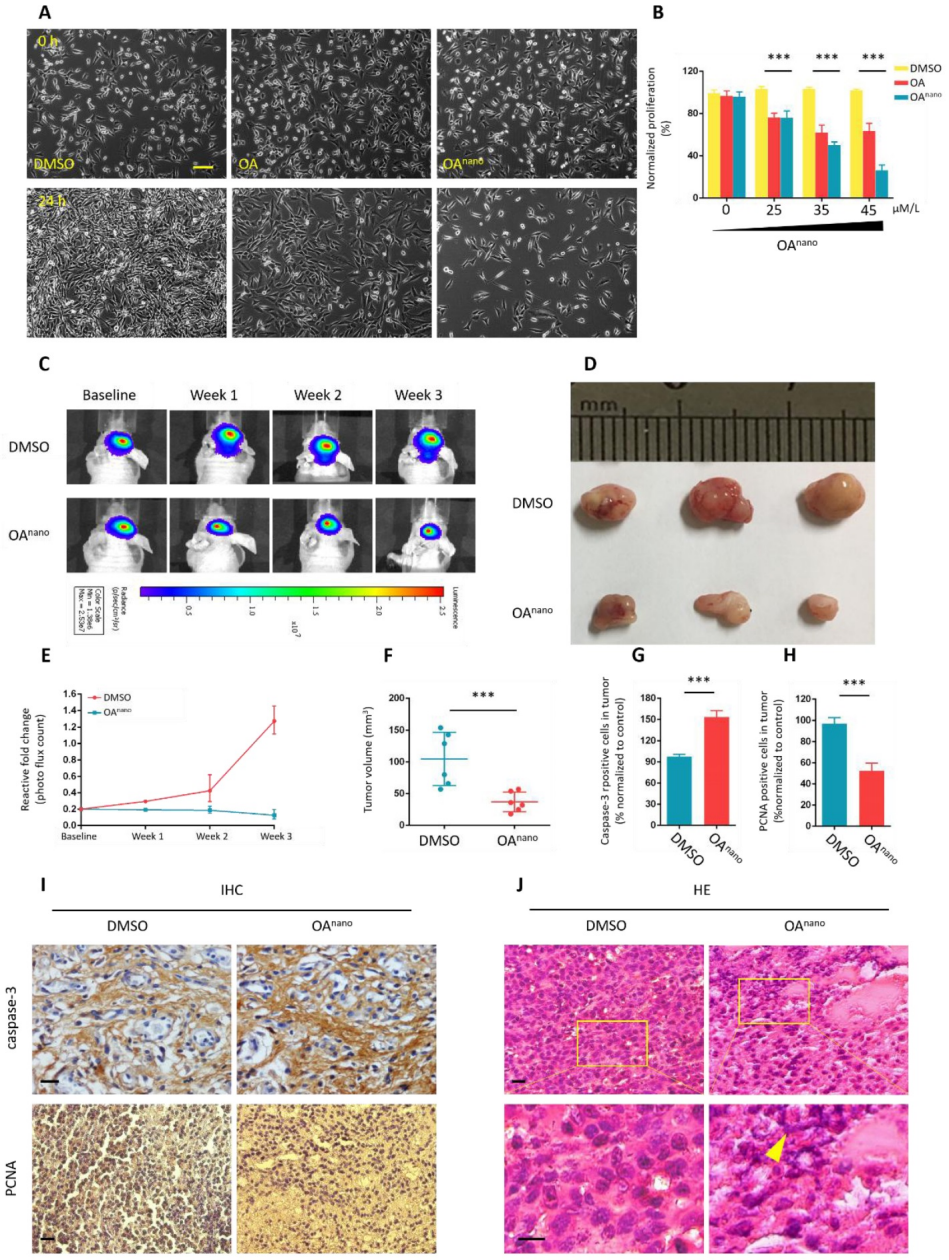
** OA^nano^ suppresses the growth of U87 glioma cells. A.** U87 glioma cells observed under a fluorescence microscope after 24 h of treatment with DMSO, DMSO-OA and DMSO-OA^nano^ at 35 μmol/L. bar=100 μm **B.** Normalized proliferation rate of U87 glioma cells after 24 h of treatment with DMSO, DMSO-OA and DMSO-OA^nano^ at different concentrations (0, 25, 35, and 45 μmol/L). **C.** Bioluminescence intensities and areas of the orthotopic glioblastoma xenograft mouse model after treatment by OA^nano^ and DMSO for 1, 2, and 3 weeks. **D.** Tumors excised from the survived mice in OA^nano^-treated and DMSO groups. **E.** Reactive fold-change of bioluminescence intensities and areas of the orthotopic glioblastoma xenograft mouse model after treatment by OA^nano^ and DMSO for 1, 2, and 3 weeks. **F.** Volume of tumors excised from the survived mice in OA^nano^-treated and DMSO groups. **G.** Caspase-3 positive cells of tumors excised from the survived mice in OA^nano^-treated and DMSO groups. **H.** PCNA positive cells of tumors excised from the survived mice in OA^nano^-treated and DMSO groups. **I.** IHC analysis of Caspase-3 and PCNA in tumors excised from the survived mice in OA^nano^-treated and DMSO groups. bar=100 μm. **J.** H&E staining of tumors excised from the survived mice in OA^nano^-treated and DMSO groups. bar=100 μm.

In the MTT assay, pronounced changes in cell morphology was observed after 24 h of OA^nano^ treatment at 45 μmol/L, and the majority of cells turning spherical with less amount of protrusions (Fig. 2-A). The number of cells also decreased in a dose-dependent manner (Fig 2-B). In contrast, in cells treated with normal OA, a limited number of those cells showed morphological changes at 45 μmol/L for 24 h, which had no statistical significance compared with control group.

Until this stage, we have verified the potent anti-tumor activity of OA^nano^ on U87 glioma cells *in vitro*, and we next established an orthotopic glioblastoma xenograft mouse model to assess its therapeutic potential *in vivo*. The model consisted of nude mice which bore U87-luciferase-expressing (U87-Luc) xenograft tumors, and were treated with OA^nano^ through daily intravenous injection for 5 consecutive days. Bioluminescence imaging was used to monitor tumor growth, and that was recorded on the 1st, 2nd, and 3rd weeks after injection.

As revealed by bioluminescence intensities and areas, the tumor size was nearly the same on the baseline, while was substantially reduced on the 2nd and 3rd weeks in OA^nano^-treated group compared with control group (Fig. 2-C, 2-E). During the experiment, 7 mice died in the OA-treated group and 14 in the control group, raising the survival rate by 100%. On the 3rd week, tumors of the survived mice were excised to calculate volume of tumors (Fig. 2-D). Upon OA^nano^ treatment, the size of tumor decreased by 72% (Fig. 2-D, 2-F) compared with the control group.

Hematoxylin and eosin (H&E) staining showed that after treatment with OA^nano^, the distribution of tumor cells became uneven with nuclei sizes remarkable varying among different cells. Furthermore, karyopyknosis, occurring during cell necrosis or apoptosis, was prominently noted in the xenograft tissues (Fig. 2-J).

Immunohistochemistry (IHC) analysis showed that OA^nano^ also induced a decrease in the number of PCNA-positive cells, while an increase in Caspase-3-positive cells was observed in comparison with control group (Fig. 2-I, 2-G, 2-H), further validating the proliferation-inhibiting and apoptosis-promoting effects of OA^nano^ *in vivo*.

In this study, 17.5 mM/kg is a relatively safe concentration, in which OA is susceptible to elicit toxicity in mice 10. In addition, the body weight of mice remained largely comparable with the control group, which could be another proof for non-systematic toxicity of OA^nano^ at the mentioned concentration. To evaluate the potential toxicity of OA^nano^ *in vivo*, we further performed H&E staining on major tissues obtained from OA^nano^-treated mice. OA^nano^ treatment did not elicit overt cellular and tissue destruction in the liver, kidney, and spleen (Figure S1A, B and C).

### OA^nano^ suppresses proliferation and promotes apoptosis of U87 glioma cells

**Figure 3:**
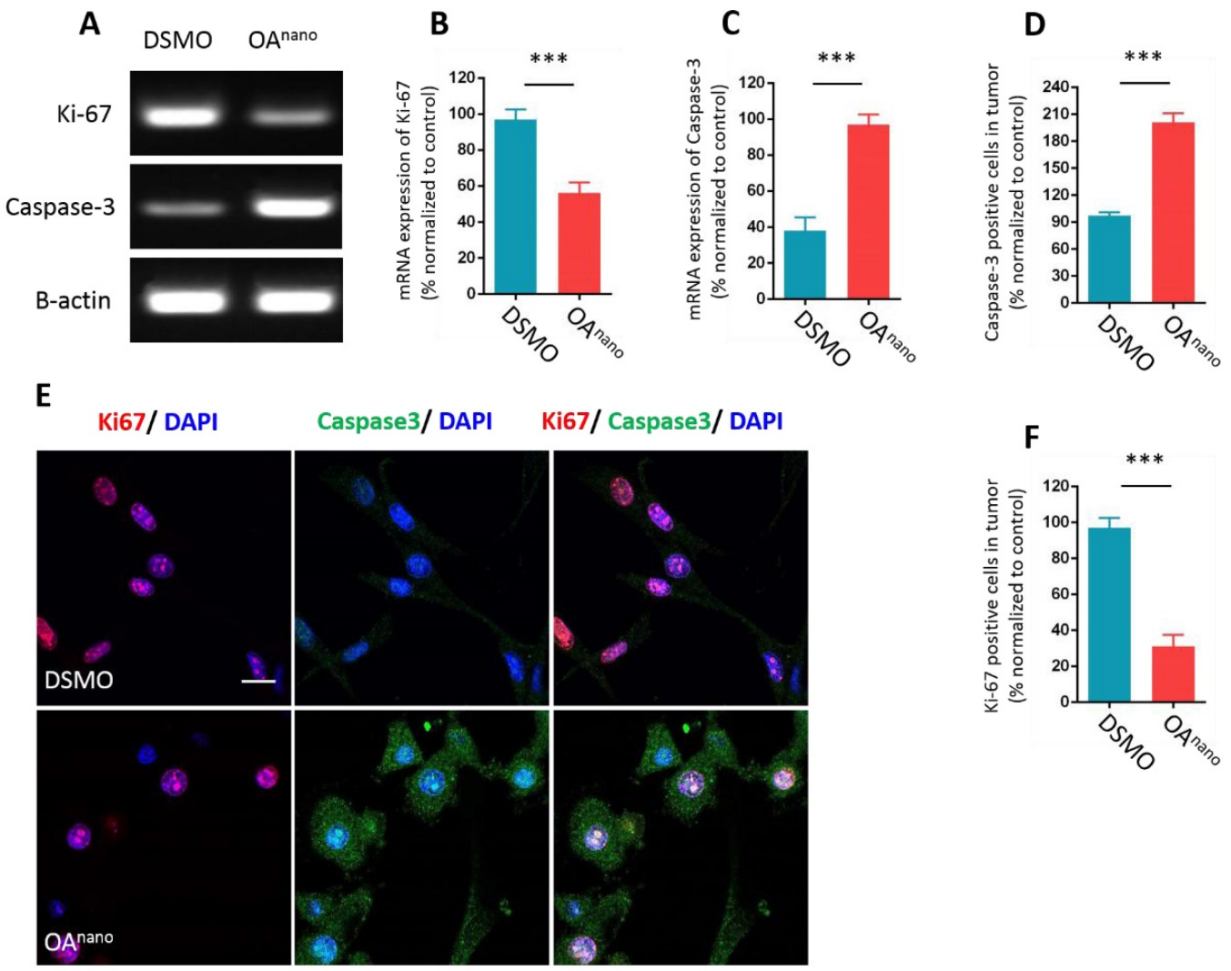
** OA^nano^ suppresses proliferation and promotes apoptosis of U87 glioma cells. A.** RT-PCR result of Ki-67 and Caspase-3 in U87 glioma cells treated with DMSO or OA^nano^ for 24 h. **B.** mRNA expression of Ki-67 in U87 glioma cells treated with DMSO or OA^nano^ for 24 h. **C.** mRNA expression of Caspase-3 in U87 glioma cells treated with DMSO or OA^nano^ for 24 h. **D.** The proportion of Caspase-3 positive U87 cells in immunofluorescence after 24 h of treatment with DMSO or OA^nano^. **E.** Immunofluorescence of Ki-67 and Caspase-3 in U87 glioma cells treated with DMSO or OA^nano^ for 24 h. bar=50 μm. **F.** The proportion of Ki-67 positive U87 glioma cells in immunofluorescence after 24 h of treatment with DMSO or OA^nano^.

To further probe the anti-tumor mechanism of OA^nano^ at 35 μmol/L, we first estimated the influence of OA^nano^ on gene expression of U87 glioma cells at mRNA level by reverse transcription polymerase chain reaction (RT-PCR). The results revealed that cells treated with OA^nano^ for 24 h significantly expressed higher amount of Caspase-3 and lower amount of Ki-67 compared with the control group (Fig. 3-A, 3-B, 3-C). Immunofluorescence (IFC) demonstrated that the number of Ki-67 positive cells was reduced by 67% after treatment with OA^nano^ for 24 h (Fig. 3-E, 3-F), and the number of Capsase-3 positive cells was 2-fold higher than the control group (Fig. 3-E, 3-D). Thus, OA^nano^ exerted an anti-tumor activity via inhibiting cell proliferation, as well as promoting cell apoptosis.

### OA^nano^ hinders migration and invasion abilities of U87 glioma cells

**Figure 4:**
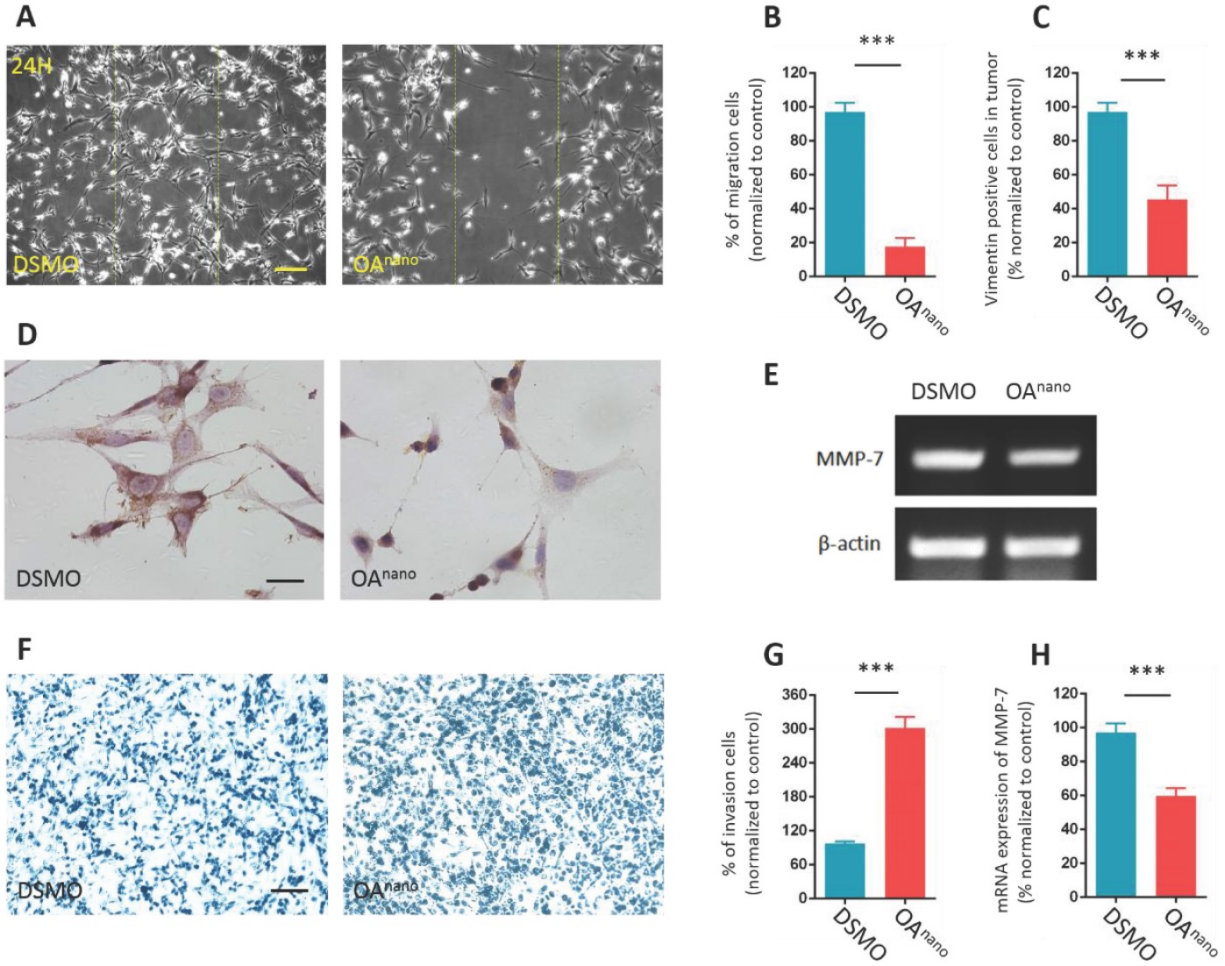
** OA^nano^ hinders migration and invasion abilities of U87 glioma cells. A.** U87 glioma cells observed under a phase contrast microscope after scratched and treated with DMSO or OA^nano^ for 24 h. The line confines the initial area of removed cells. bar=100 μm. **B.** The proportion of migrated U87 glioma cells after scratched and treated with DMSO or OA^nano^ for 24 h. **C.** The proportion of Vimentin positive U87 cells after treated with DMSO or OA^nano^ for 24 h. **D.** IHC analysis of Vimentin in U87 glioma cells after 24 h of treatment with DMSO or OA^nano^. bar=50 μm. **E.** RT-PCR analysis of MMP-7 in U87 glioma cells treated with DMSO or OA^nano^ for 24 h. **F.** U87 glioma cells which remained on the upper layer of the Transwell chamber after treated with DMSO or OA^nano^ for 24 h. bar=100 μm. **G.** The proportion of U87 glioma cells remaining on the upper membrane of the Transwell chamber after treating with DMSO or OA^nano^ for 24 h. **H.** mRNA expression of MMP-7 in U87 glioma cells after treated with DMSO or OA^nano^ for 24 h.

In this study, we carried out wound-healing assay to indicate how OA^nano^ influence on cell migration. The results disclosed that the average healing rate of cells treated with OA^nano^ for 24 h in the denuded area was reduced by 81.2% compared with the control group (Fig. 4-A, 4-B), which indicated that OA^nano^ could hinder the migration of U87 glioma cells.

Vimentin is a member of the intermediate filament family of cytoskeletal proteins and has a strong correlation with cell migration. The results of IHC analysis revealed that cells treated with OA^nano^ for 24 h transmitted Vimentin positive signals, which were more around the nuclear than in the cytoplasm (Fig. 4-C, 4-D). The impediment of cell migration and changes in cell morphology may arise from improper distribution of Vimentin.

Furthermore, crystal violet staining revealed that, in Transwell-chamber assay, the mean number of cells remained on the upper membrane in the treatment group was 3-fold higher than the control group (Fig, 4-F, 4-G), which indicated that the invasion of U87 glioma cells was hindered by OA^nano^. This finding was consistent with our RT-PCR result of down-regulation of MMP-7, as a key cancer-associated gene. After 24 h of treatment with OA^nano^, U87 glioma cells expressed about 40% less MMP-7 mRNA compared with the control group (Fig 4-E, 4-H). As a result, OA^nano^ impeded the migration and invasion of U87 glioma cells through inhibiting MMP-7 and disrupting formation and distribution of cytoskeletal protein.

## Discussion

With the overwhelming of proliferation and diffuse infiltration of healthy surrounding tissues, malignant glioma often fails to be removed by surgical resection and current chemotherapeutic approaches are not effective enough, resulting in the recurrence and dismal prognosis 2, 3.

OA is a type of traditional Chinese medicine and several scholars have studied its potential inhibitory effect on different tumors 9. However, restrained by its high molecular mass and low solubility, it is difficult for OA to reach an effective concentration in tumor tissues without excessive hepatic or renal toxicity 10. Thus, clinical researches concerning application of OA in glioma cells are limited.

Nanomedicine has proved to compensate for the limitations of traditional tumor therapies, such as improving their solubility or lipophilicity 12, 15, 16. Thus, it has a great potential to become an advanced delivery system for OA. In the present study, we attempted to cover OA with nanoparticles, as an applied drug delivery system. Using SEM, OA^nano^ was observed as round particles with uniform size and complete membrane. The oil-water partition coefficient indicated a higher lipophilicity of OA^nano^ than its normal form. Inhibitory effect of OA^nano^ on U87 glioma cells was verified *in vitro* by MTT assay and *in vivo* by constructing orthotopic glioblastoma xenograft mouse model. Those results clarified that nano-particles would not hinder the therapeutic activities of OA and more notably, OA^nano^ could impede intracranial glioma tumors, which was an attractive feature of new drug delivery systems, especially for brain tumors.

It has been reported that in *in vivo* experiment, Doxorubicin, a traditional chemotherapy drug, could significantly reduce the body weight of mice. However, in our research, the body weight and survival rate of mice remained largely comparable with the control group and H&E staining on major tissues obtained from OA^nano^-treated mice showed no obvious cellular and tissue destruction, which suggest low systematic side effect was exerted by OA^nano^. This can be illustrated by the enhanced permeability retention effect of liposome. As for the potential systematic toxicity of high dosage of OA^nano^, Joana A Loureiro *et al* 17 found liposome modified with antibody can increase concentration of drug in tumor so that the dosage along with the incidence of side effect can be reduced.

Targeted therapy of nanoliposomes has certain limitations and is easily ingested by mononuclear macrophage system (RES) located in liver, spleen, lung and bone marrow. Mohamadreza Amin *et al* 18 found that increasing the peptide hydrophobicity promotes the therapeutic efficacy of RGD-SSLD in a C-26 tumor model due to decreased recognition by RES. In our study, no significant intake by RES was observed in the H&E staining on liver obtained from OA^nano^-treated mice. This may be associated with the larger size of nanoliposome (55 nm as average) than traditional one.

To yield an integrated view on the mechanisms in which OA^nano^ influences U87 glioma cells, RT-PCR, IHC, and other methods were conducted on U87 glioma cells with or without OA^nano^ treatment. The findings could facilitate the understanding of the molecular mechanisms of drugs, the pharmacodynamic markers of drug response, and the gene expression patterns that account for drug sensitivity and resistance.

Expectedly, U87 glioma cells treated with OA^nano^ were tested to have down-regulation and up-regulation effects on Ki-67 and Caspase-3, respectively, and it was revealed that OA^nano^ exerted its activities through inhibiting proliferation and promoting apoptosis of U87 glioma cells. Moreover, a notable reduction of Vimentin and MMP-7 was noted, which contributed to the round shape and less protrusions in U87 glioma cells after treatment, which eventually led to the disability of migration and invasion.

Ki-67 is one of the most commonly used proliferation markers in clinical pathology and diagnosis. Melike *et al*. detected a significant reduction in the number of Ki-67 positive glioma C6 cells treated with hesperetin 10. Apoptosis refers to the process of programmed cell death that may occur in multicellular organisms. The apoptotic pathway is activated by both intracellular and extracellular signals. There are two different pathways that lead to apoptosis: the intrinsic and extrinsic pathways that correlate with the signal type. They are also referred to as the mitochondrial and death receptor pathways, respectively. Both pathways can initiate caspase family proteins, including Caspase-3, Caspase-7, Caspase-8, etc. These proteins activate deoxyribonuclease, leading to chromosomal DNA fragmentation during apoptosis 19, 20.

Quintavalle *et al*. investigated the effects of miR-21 and miR-30 a/b on glioma and found that it promoted apoptosis of glioma cells by increasing the expression level of Caspase-3 21. A similar phenomenon was observed in Chang *et al*.'s experiment; in their study, when resveratrol was added into U87 glioma cells, and the positive expression rate of Caspase-3 was significantly increased, which caused cell apoptosis 22. In the present study, it was revealed that, due to anti-tumor mechanism of OA^nano^, it suppressed proliferation and promoted apoptosis of U87 glioma cells by down-regulating the expression level of Ki-67 and up-regulating the expression level of Caspase-3.

Tumor metastasis is a key factor causing to an increase in the tumor malignancy 3. The ability to migrate in movable cells (e.g., platelets and osteoclasts) is highly correlated with the cytoskeleton protein inside the cells 23. When the expression level of Vimentin in tumor cells is increased and overwhelming keratin as the main component of the cytoskeleton, epithelial-mesenchymal transition (EMT) occurs, playing a substantial role in tumor invasion and metastasis 24. Thus, an abnormally elevated expression of Vimentin is considered as a marker for enhanced invasive ability of cancer cells. Miklossy *et al*. found that hirsutinolide inhibited the migration of human glioma cells, and also detected a significant decrease in the expression level of Vimentin 25. Guo *et al* reported that OA drugs are closely related to MAPK/ERK signaling pathway in the tumorigenesis mechanism of glioma *in vitro* 24. Similar points are shared in this study by revealing the down-regulation of Vimentin, the down streaming production of MAPK/ERK pathway, and the consequently destruction of cytoskeleton. In addition, by covered with nanoliposomes, the physical limitation of OA concentration was overcome and the results were further proved in *in vivo* experiment. Matrix metalloproteinase (MMP) can degrade the extracellular matrix and expand the space for rapid tumor growth and ultimately lead to tumor metastasis. A previous study revealed that degradation of bone matrix exerted by osteoclasts is associated with MMPs 26. MMP-7 is one of the MMP families, and that has been proved to participate in the degradation of laminin and type IV collagen 27, 28. In study of brain glioma tissues, Rome *et al*. found that the pathological grades of glioma were positively correlated with the expression level of MMP-7 and might be associated with activation of ERK1/2 signaling pathway 27. In the current study, IHC analysis of Vimentin and RT-PCR detection of MMP-7 showed that the expression levels of Vimentin and MMP-7 in U87 glioma cells were decreased in OA^nano^ group, suggesting that OA impeded the migration and invasion of U87 glioma cells.

To sum up, through *in vitro* and *in vivo* experiments, the following conclusions can be drawn: The solubility and lipophilicity of OA were improved after covering with nanoliposomes; OA^nano^ suppressed proliferation, induced apoptosis and degraded migration ability of glioma cells, which also had an anticancer effect on glioma found to be better than that of OA. Nevertheless, an appropriate dosage or administration route of OA^nano^ in clinical practice needs to be further studied. Our results may provide theoretical and experimental basis for development of new drugs for treatment of glioma in the future.

## Supplementary Material

Supplementary figures and tables.

## Abbreviations

OA: oleanolic acid
OA^nano^: oleanolic acid covered with polymer nano-particles
DMSO: dimethyl sulfoxide

## References

[1] Etminan N, Peters C, Ficnar J, Anlasik S, Bunemann E, Slotty PJ, Hanggi D, Steiger HJ, Sorg RV, Stummer W (2011). Modulation of migratory activity and invasiveness of human glioma spheroids following 5-aminolevulinic acid-based photodynamic treatment. Laboratory investigation. J Neurosurg.

[2] Chen M, Dai E (2006). Tumour stem cell-targeted treatment: elimination or differentiation. Oncology.

[3] Maugeri-Sacca M, Vigneri P, De Maria R (2011). Cancer stem cells and chemosensitivity. Clin Cancer Res.

[4] Liu Z, Li H, He L, Xiang Y, Tian C, Li C, Tan P, Jing J, Tian Y, Du L and (2019). Discovery of Small-Molecule Inhibitors of the HSP90-Calcineurin-NFAT Pathway against Glioblastoma. Cell Chem Biol.

[5] Meng KK (2007). Tumorigenesis of chemotherapeutic drug-resistant cancer stem-like cells in brain glioma. Cells and Development.

[6] Pan BG, Tang T, Gang J, Chen K (2009). Identification of selective inhibitors of cancer stem cells by high-throughput screening. Cell.

[7] Scopelliti A, Cammareri P, Catalano V, Saladino V, Todaro M, Stassi G (2009). Therapeutic implications of Cancer Initiating Cells. Expert Opin Biol Ther.

[8] Sorensen MD, Fosmark S, Hellwege S, Beier D, Kristensen BW, Beier CP (2015) (2015). Chemoresistance and chemotherapy targeting stem-like cells in malignant glioma. Adv Exp Med Biol.

[9] Abdelmageed N, Morad SA, Elghoneimy AA, Syrovets T, Simmet T, El-Zorba H, El-Banna HA, Cabot M, Abdel-Aziz MI (2017). Oleanolic acid methyl ester, a novel cytotoxic mitocan, induces cell cycle arrest and ROS-Mediated cell death in castration-resistant prostate cancer PC-3 cells. Biomed Pharmacother.

[10] Ma W, Wang DD, Li L, Feng YK, Gu HM, Zhu GM, Piao JH, Yang Y, Gao X, Zhang PX (2014). Caveolin-1 plays a key role in the oleanolic acid-induced apoptosis of HL-60 cells. Oncol Rep.

[11] Meola A, Rao J, Chaudhary N, Sharma M, Chang SD (2018). Gold Nanoparticles for Brain Tumor Imaging: A Systematic Review. Front Neurol.

[12] Tomas S, Milanesi L (2009). Hydrophobically self-assembled nanoparticles as molecular receptors in water. J Am Chem Soc.

[13] Zhu Z (2014). Flash nanoprecipitation: prediction and enhancement of particle stability via drug structure. Mol Pharm.

[14] Ran H (2006). Polymer therapeutics: concepts and applications. Chemie.

[15] Yu SC, Yi L, Zhou ZH, Yao XH, Ping YF, Bian XW (2007). Isolation and identification of tumor stem-like cells from human glioma cell line U87 after treatment of vincristine. Ai Zheng.

[16] Wang Y, Zhu P, Li G, Zhu S, Liu K, Liu Y, He J, Lei J (2019). Amphiphilic carboxylated cellulose-g-poly(l-lactide) copolymer nanoparticles for oleanolic acid delivery. Carbohydr Polym.

[17] Loureiro JA, Gomes B, Coelho MA, do Carmo Pereira M (2014). Targeting nanoparticles across the blood-brain barrier with monoclonal antibodies. Nanomedicine.

[18] Amin M, Mansourian M, Koning GA, Badiee A, Jaafari MR (2015). Development of a novel cyclic RGD peptide for multiple targeting approaches of liposomes to tumor region. Journal of Controlled Release.

[19] Peng XP, Li XH, Li Y, Huang XT, Luo ZQ (2019). The protective effect of oleanolic acid on NMDA-induced MLE-12 cells apoptosis and lung injury in mice by activating SIRT1 and reducing NF-kappaB acetylation. Int Immunopharmacol.

[20] Ersoz M, Erdemir A, Duranoglu D, Uzunoglu D, Arasoglu T, Derman S, Mansuroglu B (2019). Comparative evaluation of hesperetin loaded nanoparticles for anticancer activity against C6 glioma cancer cells. Artif Cells Nanomed Biotechnol.

[21] Quintavalle C, Mangani D, Roscigno G, Romano G, Diaz-Lagares A, Iaboni M, Donnarumma E and (2013). MiR-221/222 target the DNA methyltransferase MGMT in glioma cells. PLoS ONE.

[22] Zhang R, Wang R, Chang H, Wu F, Liu C, Deng D, Fan W (2012). Downregulation of Ezh2 expression by RNA interference induces cell cycle arrest in the G0/G1 phase and apoptosis in U87 human glioma cells. Oncol Rep.

[23] Grindel BJ, Martinez JR, Tellman TV, Harrington DA, Zafar H, Nakhleh L, Chung LW, Farach-Carson MC (2018). Matrilysin/MMP-7 Cleavage of Perlecan/HSPG2 Complexed with Semaphorin 3A Supports FAK-Mediated Stromal Invasion by Prostate Cancer Cells. Sci Rep.

[24] Guo G, Yao W, Zhang Q, Bo Y (2013). Oleanolic acid suppresses migration and invasion of malignant glioma cells by inactivating MAPK/ERK signaling pathway. Plos One.

[25] Miklossy G, Youn U J, Yue P, Zhang M, Chen C H, Hilliard T S, Paladino D, Li Y, Choi J, Sarkaria J N (2015). Hirsutinolide Series Inhibit Stat3 Activity, Alter GCN1, MAP1B, Hsp105, G6PD, Vimentin, TrxR1, and Importin alpha-2 Expression, and Induce Antitumor Effects against Human Glioma. J Med Chem.

[26] Grindel BJ, Martinez JR, Pennington CL, Muldoon M, Stave J, Chung LW, Farach-Carson MC (2014). Matrilysin/matrix metalloproteinase-7(MMP7) cleavage of perlecan/HSPG2 creates a molecular switch to alter prostate cancer cell behavior. Matrix Biol.

[27] Rome C, Arsaut J, Taris C, Couillaud F, Loiseau H (2007). MMP-7 (matrilysin) expression in human brain tumors. Mol Carcinog.

[28] Zhang H, Wang Y, Chen T, Zhang Y, Xu R, Wang W, Cheng M, Chen Q (2019). Aberrant Activation Of Hedgehog Signalling Promotes Cell Migration And Invasion Via Matrix Metalloproteinase-7 In Ovarian Cancer Cells. J Cancer.

