# Supplementary material for: Nanocrystallized Oleanolic Acid Better Inhibits Proliferation, Migration and Invasion in Intracranial Glioma via Caspase-3 Pathway
Source: J Cancer. 2020 Jan 29;11(7):1949–58. doi: 10.7150/jca.38847 (PMC7052863; doi:10.7150/jca.38847)
Supplement: Supplementary file 1 — Supplementary figures and tables. [file jcav11p1949s1.pdf]

Supplementary Figures

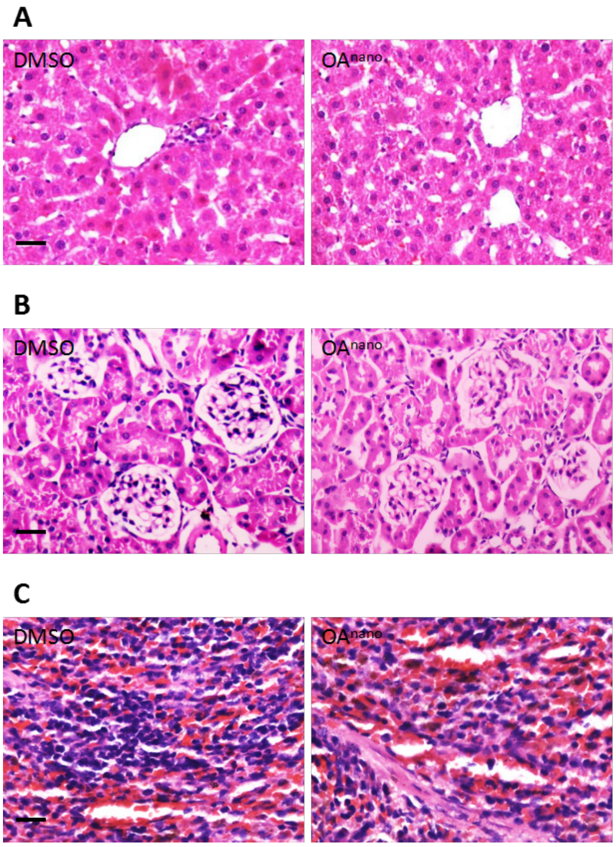

Supplementary figures OA^{nano} treatment did not elicit overt cellular and tissue destruction in the liver, kidney and spleen

A: H&E staining of liver obtained from nude mice after treatment by OA^{nano} and DMSO for 3 weeks.

B: H&E staining of kidney obtained from nude mice after treatment by OA^{nano} and DMSO for 3 weeks.

C: H&E staining of spleen obtained from nude mice after treatment by OA^{nano} and DMSO for 3 weeks.
